# Research on the forward-looking behavior judgment of heating oil price evolution based on complex networks

**DOI:** 10.1371/journal.pone.0202209

**Published:** 2018-09-05

**Authors:** Lixin Tian, Huan Chen, Zaili Zhen

**Affiliations:** 1 Energy Development and Environmental Protection Strategy Research Center, Jiangsu University, Zhenjiang, Jiangsu, China; 2 Energy Interdependency Behavior and Strategy Research Center, School of Mathematical Science, Nanjing Normal University, Nanjing, Jiangsu, China; Central South University, CHINA

## Abstract

Analyzing and predicting the trend of price fluctuation has been receiving more and more attention, as price risk has become the focus of risk control research in heating oil futures market. A novel time series prediction model combined with the complex network method is put forward in the paper. First of all, this paper counts the cumulative time interval of different nodes in the network, and fits its growth trend with the Fourier model. Then a novel price fluctuation prediction model is established based on the effective information such as some topology properties extracted from the network. The results show that the Fourier model can predict the emergence time of new nodes in the next stage, and the established price fluctuation prediction model can infer the names of nodes in the prediction interval, so as to determine the forward-looking behavior of price evolution. Besides, liken to the NAR neural network, the prediction results obtained by the proposed method also show superiority, which has important theoretical value and academic significance for early warning and prediction of price behavior in the heating oil futures market.

## Introduction

Time series is an emergent output of a system that contains much information about its microscopic details, while time series prediction plays an active role in the field of science and engineering, it refers to the level that can be predicted for the next period of time according to the development process, direction, and trend reflected by time series to make analogies or extensions, which is based on the establishment and analysis of the time series. Since data forecasting is universal in every field of life, the research of time series forecasting technology has profound application value and theoretical significance. In recent years, there has been a large body of literature exploring new and complex forecasting techniques, which promotes the study and use of linear models. For instance, Gooijer and Hyndman [1] reviewed the past 25 years of research into time series forecasting, which had promoted the study and use of linear models such as the autoregressive (AR), moving averages (MA), autoregressive moving averages (ARMA) and autoregressive integrated moving averages (ARIMA), and so on. Cuaresma etc. [2] studied the forecasting abilities of a battery of univariate models on hourly electricity spot prices using AR, ARMA models and unobserved components models.

In fact, many systems usually have unknown nonlinear structures [3,4]. The development of nonlinear time series is still in a period of preliminary exploration in terms of its analysis and prediction when compared with the study of linear time series, but in recent years it has become a hot topic. The nonlinear methods, which are commonly used in the prediction field, are neural networks (including Back Propagation (BP) neural network, Radial Basis Function (RBF) neural network, Elman neural network, NAR neural network, Recursive Delay Network, etc.) [5–10], Support Vector Machine (SVM) [11], Chaos Theory [12], and Combination Forecast [13–18]. Among them, the Combination Forecast method gathers some useful information of individual forecasting methods, which can improve the prediction accuracy and reduce the prediction risk, thus it has been widely used in recent years. Hu et al. [19] proposed a hybrid forecasting approach combined the Ensemble Empirical Mode Decomposition (EEMD) and the Support Vector Machine (SVM), which was used to improve the quality of wind speed forecasting. Adhikari [20] presented a linear combination method for time series forecasting that determined the combining weights through a novel neural network structure, it had been verified that this combined forecast improved the overall accuracy to a great extent and was often better than the forecast of each component model. Zhang et al. [21] put forward a novel weighted combination method, namely ARIMA-WASDN method (ARIMA means autoregressive integrated moving average, and WASDN means weights and structure determination network), so as to improve the forecasting accuracy and enhance the applicability of the time series forecasting approach.

Studies over the past few years have shown that a system can be abstracted into one or more complex networks if there are a large number of structural units inside and interacting with each other, which makes the complex network, a powerful research tool, have achieved remarkable development in complex systems of many fields. Jovani and Fortuna [22] studied some information about the past events by analyzing the frequency of occurrence of numbers associated with years in the World Wide Web. Williams and Martinez [23] showed that a remarkably simple model by successfully predicting key structural properties of the most complex and comprehensive food webs in the primary literature. In addition, common networks include communication networks, electric power grid, bus transport network, oscillator networks, traffic flow networks, and so on [24–29]. So far, many scholars have tried to combine the application of network theory with time series analysis, that is, it is expected that new structural features can be extracted to gain insight into the structural and dynamic characteristics of complex networks by constantly exploring the methods of mapping from time series to complex networks. Gao et al. [30] constructed a directed weighted complex network (DWCN) from a time series using a heuristic theory for determining the threshold, then associated different aspects of chaotic dynamics with the topological indices of DWCN and demonstrated that DWCN can be exploited to detect unstable periodic orbits of low periods. Wang et al. [31] developed a simple and fast computational method which utilized the phase space coarse graining algorithm that converted a time series into a directed and weighted complex network. Thereby, they found that the phase space coarse graining algorithm could allow people to distinguish, identify and describevarious time series in detail. Ferreira et al. [32] pointed out a method to transform a set of time series into a network using different distance functions, where each time series was represented by a vertex and the most similar ones were connected, then applied community detection algorithms to identify groups of strongly connected vertices. Experimental study showed that the proposed network-based approach achieved better results than various classic or up-to-date clustering techniques under consideration.

**Fig 1:**
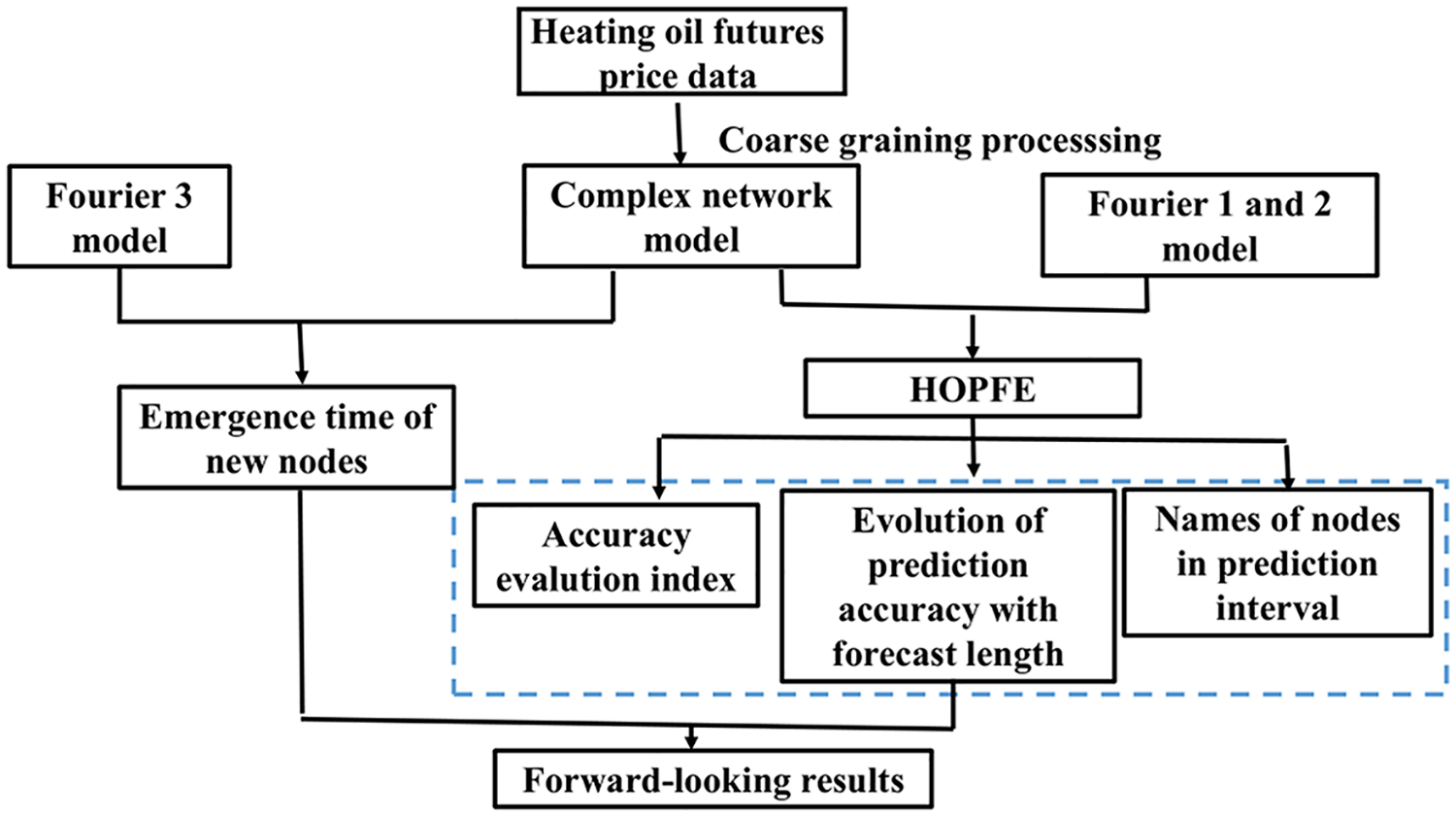
The overall research framework chart.

The implementation of price fluctuation forecasting and the popularity of various linear models have been the focus of attention in the literature of energy market prices over the past decade [33], the most commonly used predictive methods of energy price volatility are the short-term forward curve, generalized autoregressive conditional heteroskedasticity (GARCH) model and its related neural network hybrid models [34]. Previous studies used to take advantage of complex network methods to study energy price series, but rarely involve the prediction of price volatility. In addition, a large number of existing literature often use statistical and econometric models to quantitatively analyze the correlations between energy and market risks [35–37]. Considering that there are potentially different periods of fluctuations in energy prices, examining the topological dynamics of price fluctuations over time will provide more reference for price decision makers, while complex networks are great tools to study network topology. From this point of view, this paper presents a new time series forecasting method based on complex networks, and there is research on the futures price volatility forecast of American heating oil as a case study. What’s more, it judges the forward-looking behavior of short-term futures price fluctuation. The research framework of this paper is shown in Fig 1. First of all, constructing the complex network model and analyzing the transformation characteristics between price fluctuation modes based on the results of coarse-grained treatment of heating oil futures prices from June 1, 2001 to June 10, 2016. Secondly, the cumulative time interval of different nodes in the network is calculated, with its growth trend fitted by Fourier model [38], thereby predicting the emergence time of new nodes in the next stage. At the same time, using the effective information such as some topological properties extracted from the network to identify the transformation relationship between the mentioned 15-year fluctuation modes, then the prediction model of heating oil prices (HOPFM) is established. Next, we forecast the ups and downs of heating oil prices covering the period from May 6, 2016 to June 12, 2016, and further infer the names of nodes in forecast interval. For another, comparing the predicted results with those obtained from the NAR neural network model, given the NAR neural network is a powerful computational model for modeling and predicting nonlinear time series, it can further illustrate the superiority of the model established in our paper.

## Data and methods

### Sources and processing of data

**Fig 2:**
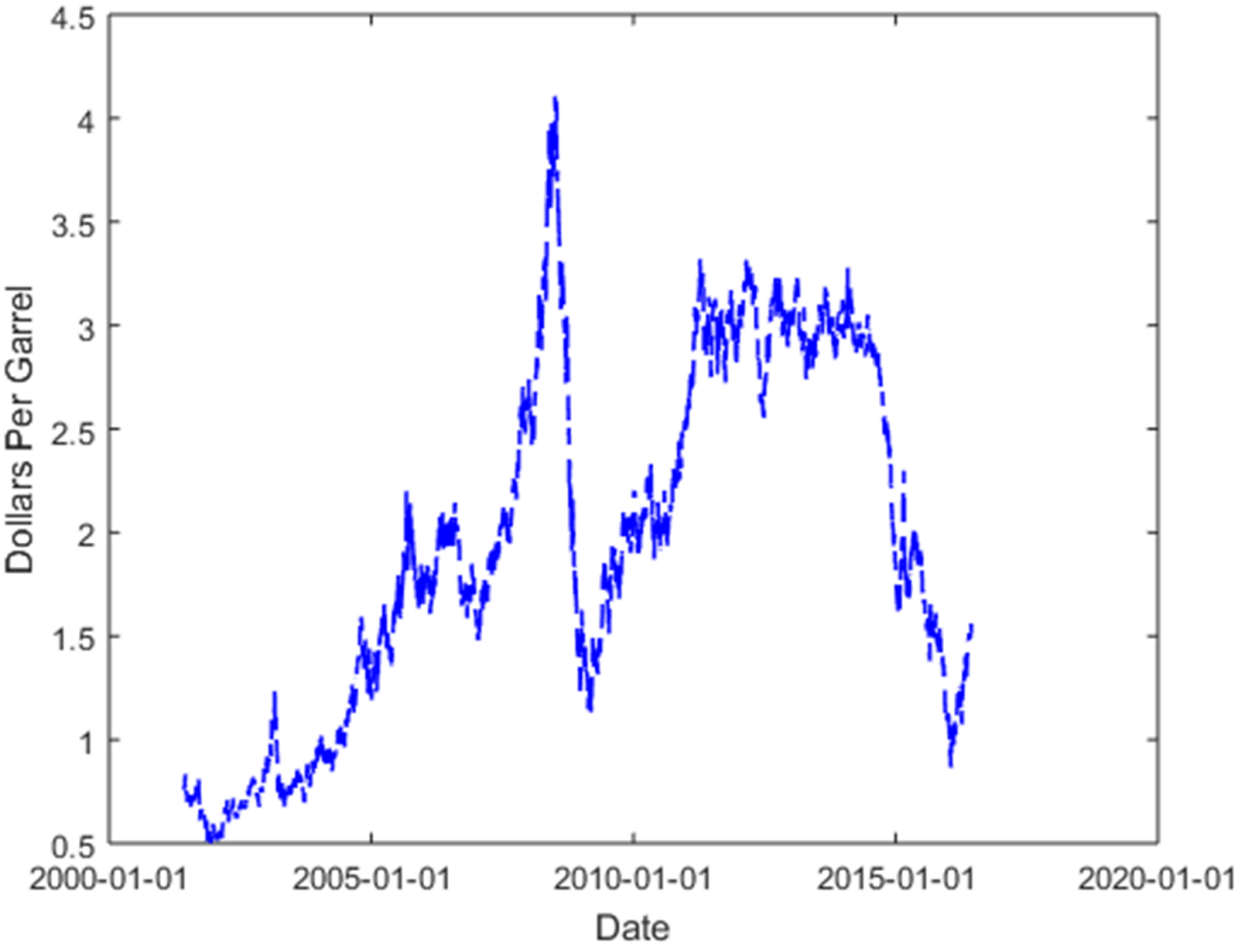
Futures price trend of heating oil.

The data selected in this article are from New York Harbor No. 2 Heating Oil Future Contract 1 (Dollars per Gallon) covering the period from June 1, 2001 to June 10, 2016, which can be downloaded from US Energy Information Administration (https://www.eia.gov/dnav/pet/hist/LeafHandler.ashx?n=PET&s=EER_EPD2F_PE1_Y35NY_DPG&f=D), this 15-year period generates a total of 3765 prices data, and their fluctuation trend is shown in Fig 2.

First of all, the coarse graining algorithm [39] is taken to process these data, and the futures price series of heating oil are denoted by *P*_*f*_(*t*)(*t* = 1, 2, 3,…,*n*,*n* = 3765). It is assumed that *P*_*f*_(*t*) represents the current price, and *P*_*f*_(*t* − 1) refers to the price of the day before *t*, then the sequence of futures price volatility is Δ*P*_*f*_(*t*) = *P*_*f*_(*t*) − *P*_*f*_(*t* − 1).

Next, we define $H_{\Delta P_{f}}=\frac{\sum_{t=1}^{n-1}\left|\Delta P_{f}\left(t\right)\right|}{n-1}$ as the average fluctuation sequence of heating oil futures prices. It means that heating oil prices rise sharply when $\Delta P_{f}>H_{\Delta P_{f}}$, and rise steadily if $0<\Delta P_{f}\leq H_{\Delta P_{f}}$. At the same time, the prices are steady when Δ*P*_*f*_ = 0, and face a steady fall if $-H_{\Delta P_{f}}\leq \Delta P_{f}<0$. Besides, it reveals a sharp fall when $\Delta P_{f}<-H_{\Delta P_{f}}$. Five characters are made use of to represent the five fluctuating states respectively in heating oil futures prices, then the price fluctuation sequence of heating oil for five working days in a week can be expressed as

$$hf_{i}=\left\{ \begin{array}{l} G,\Delta P_{f}>H_{\Delta P_{f}}\\ g,0<\Delta P_{f}\leq H_{\Delta P_{f}}\\ s,\Delta P_{f}=0\\ f,-H_{\Delta P_{f}}\leq \Delta P_{f}<0\\ F,\Delta P_{f}<-H_{\Delta P_{f}} \end{array}\right.$$ (1)

where *G*, *g*, *s*, *f*, *F* respectively denote the states of sharp rise, steady rise, stabilization, steady fall and sharp fall. According to the method mentioned above, the fluctuation sequence of heating oil futures prices can be transformed into corresponding symbol sequences

$$S=\left\{hf_{1},hf_{2},hf_{3},\cdots \right\},hf_{i}\in \left(G,g,s,f,F\right)$$ (2)

### Analysis methods

#### Complex network models

In general, the trade days of heating oil futures market are five working days in a week. Therefore, the price fluctuation status of 5 days are separately represented by 5 volatility symbols on the basis of futures price symbol sequence *S* = {*hf*_*i*_}, *i* = 1, 2, 3,⋯, 3765. Then we make them form a symbol sequence which is defined as a mode, and then choose one day for the step to do data sliding. As a consequence, 3761 futures price volatility modes are obtained. What’s more, the former mode is the basis for the formation of the latter mode due to data sliding, and it is directed and transitive for a mode [40]. Therefore, it is feasible to construct a directional weighted network of futures prices with each fluctuation mode as a node, the directional transformation between two modes as the edge and the transformation times as the weight of edges. The construction process is shown in Table 1.

**Table 1:** Construction process of a directed weighted network for the futures prices fluctuations.

**Date**	***P***_***f***_ ***(t)***	**mode**
2001-06-01	0.759	*ssgff*
2001-06-04	0.759	*sgffg*
2001-06-05	0.770	*gffgg*
2001-06-06	0.766	*ffggg*
2001-06-07	0.759	*fgggf*
2001-06-08	0.767	*gggfG*
2001-06-11	0.783	*ggfGf*
2001-06-12	0.802	*gfGfF*
**Date**	***P***_***f***_ ***(t)***	**mode**
2001-06-13	0.801	*fGfFg*
2001-06-14	0.833	*GfFgF*
2001-06-15	0.805	*fFgFs*
⋯	⋯	⋯
2016-06-03	1.488	*fgGGf*
2016-06-06	1.503	*gGGfF*

A corresponding directed weighted network is constructed on the basis of fluctuation mode sequence {*ssgff*, *sgffg*, *gffgg*, *ffggg*, *fgggf*,⋯,*gGGfF*} obtained from Table 1, where each node indicates the fluctuating state of heating oil futures prices. Some volatility modes will appear repeatedly according to the results of coarse-grained treatment, then 1202 different modes or 1202 different nodes are finally gotten after careful deletion.

#### Fourier models

Fluctuation trends of heating oil futures prices are affected by a variety of factors such as seasonal demand for heating oil, competition in local markets and regional operating costs. Among these influencing factors, some factors play a long-term and decisive role in the fluctuation of prices that can lead to a certain degree of tendency and regularity. While some other factors play a short-term and non-decisive role, resulting some irregularities in price fluctuations. These factors influence the trend of price fluctuations, which in turn affect the emergence time of new price volatility modes. On one hand, for those factors that have long-term and decisive effects, the cumulative time interval for new nodes shows an upward trend. Theoretically, a monotonically increasing function *f*(*t*) can be used to describe it. For example, the linear function *f*(*t*) = *kt* + *b*, where *k* > 0. On the other hand, some influencing factors *x*_*n*_ (*n* = 1, 2, 3…*N*) make the price fluctuation appear as seasonal or periodic, and thus the cumulative time interval at which new nodes appear can not show a linear increase because their frequency of influence may vary. Assuming that a kind of factor *x*_*n*_ affects the cumulative time of new nodes, with its influence frequency is expressed as *nw*. Then the function *a*_*n*_ cos(*nwt*) + *b*_*n*_ sin(*nwt*) can be used to describe the effect of *x*_*n*_ on cumulative time intervals for new nodes. For the sake of simplicity, it is assumed that these various influencing factors are independent of each other. Therefore, based on the above analysis and hypothesis, a trend fitting model for time series [40] can be established as follows:

$$\begin{array}{l} f(t)=a_{0}+a_{1}\text{cos}(wt)+b_{1}\text{sin}(wt)+a_{2}\text{cos}(2wt)+b_{2}\text{sin}(2wt)+\ldots +a_{n}\text{cos}(nwt)+b_{n}\text{sin}(nwt)+\ldots \\ =a_{0}+\sum_{n=1}^{\infty }\left[a_{n}\text{cos}(nwt)+b_{n}\text{sin}(nwt)\right] \end{array}$$ (3)

where *a*_0_ is the long-term development trend parameter of cumulative time intervals, and *a*_*n*_ is the periodic influence parameter of *x*_*n*_, which reflects the amplitude of influencing and the phase of cycle. If the period of *f*(*t*) is *T*, then the angular frequency $w=\frac{2\pi }{T}$, and the frequency $f=\frac{1}{T}$.

In conclusion, through the Fourier transform, the trigonometric sequence can approximate the function of a discontinuous point with arbitrary precision. This model not only solves the shortcomings of previous balance of various influencing factors, but also provides a novel method for the forecasting theory of the cumulative time interval of new nodes.

#### The forecasting model HOPFM

As can be seen from the complex network model constructed above, 3761 price fluctuation modes and 1202 new nodes can be obtained if the futures price data of heating oil from June 1, 2001 to June 10, 2016 are selected as the research object or the training set, and these 3761 price fluctuation modes are from 1202 different nodes between the conversion of each other. In addition, since the emergence of new nodes often takes a certain amount of time, there are often some nodes appeared ever that have appeared after the emergence of a node but before the next new node. Let the original set of different nodes be *V* = {*V*_1_, *V*_2_, *V*_3_,⋯,*V*_*t*_}, then the node to be predicted at *t* + 1 is one of $V_{t+1}=\left\{V_{1}{}^{t+1},V_{2}{}^{t+1},V_{3}{}^{t+1},V_{n}{}^{t+1}\right\}$ (the number of *n* is no more than *t*). To identify exactly which node it is will be a complex problem with heavy workload, for which we design the following rules in combination with some topological properties of the price fluctuation network of heating oil.

Rule I The priority selection principle of the greatest node intensity, that is, it is preferred to select a node with the highest intensity in *V*_*t*+1_ as the node to be present at time *t* + 1;
Rule II The priority selection principle of the largest betweenness centrality, that is, a node with the largest betweenness from the set of *V*_*t*+1_ is selected as the node to appear at *t* + 1;
Rule III The principle of preferring the maximum connection probability, namely, a node from the set of *V*_*t*+1_ that is of the largest probability with *V*_*t*_ is selected as the node to be present at *t* + 1;
Rule IV The shortest geodesic (geodesic length is not zero) priority principle, it is to choose a node in *V*_*t*+1_, which is of a shortest distance with *V*_*t*_, as the node will appear at *t* + 1.

**Fig 3:**
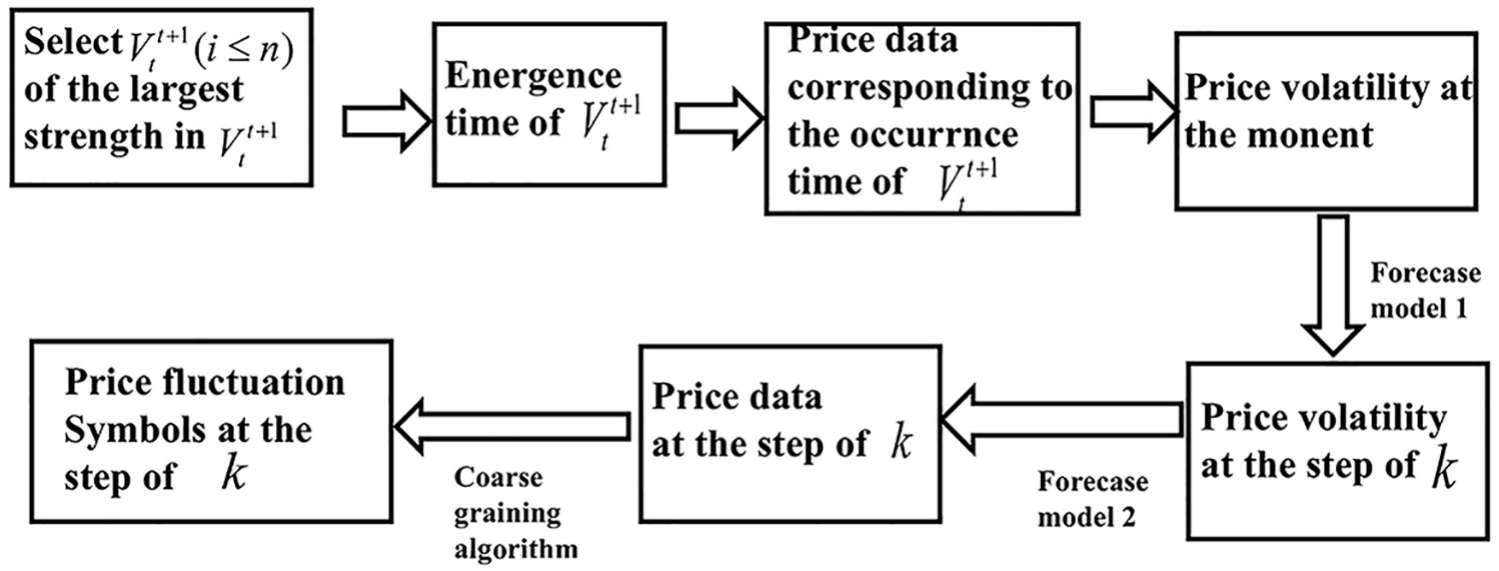
An idea flow chart.

Taking rule I as an example, the idea flow of establishing a forecasting model is shown in Fig 3:

Therefore, we give specific prediction models in the following.

Forecast mode ①

**Fig 4:**
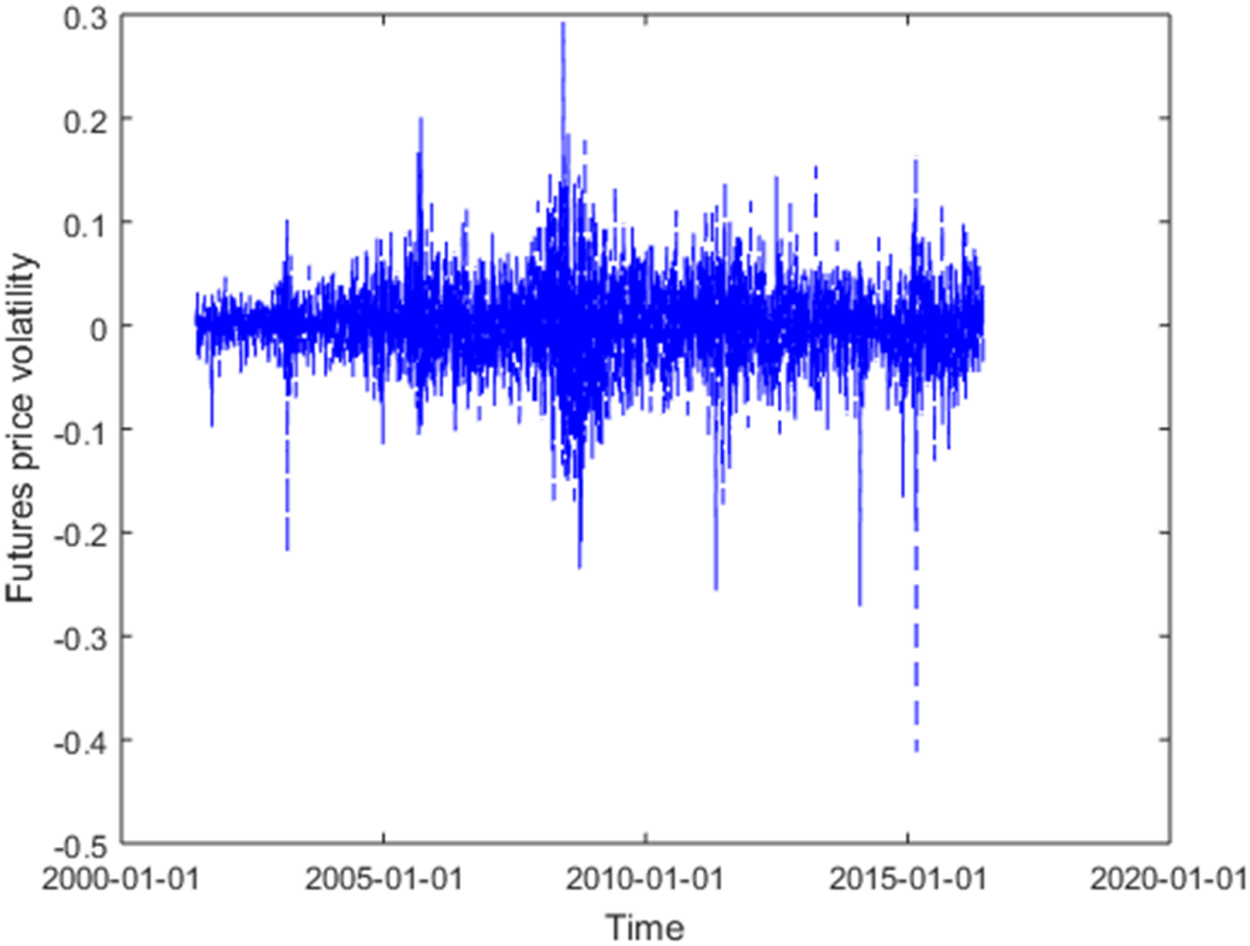
Volatility rate of heating oil futures prices in the training set.

Given all the information *F*_*t*_ = (*p*_1_, *p*_2_,⋯, *p*_*t*−1_, *p*_t_) till *t*, where *p*_*i*_ (*i* = 1, 2,⋯, *t*) is the price volatility at *i*, and the price volatility within the training set is shown in Fig 4. Assuming we would like to predict the value of price volatility sequence at the future time *T* = *t*+1, *t*+2,⋯, *t*+*K*, with *t* and *k* (*k* ∈ 1,2,…,*K*) respectively denoting the predicted starting point and the period for forward prediction, then the forward forecast of *k* period for price volatility is expressed as

$$F_{t+k}{}^{*}=\sum_{j=1}^{s}w_{j}(f(F_{i})+\varepsilon_{t})$$ (4)

As a matter of fact, *F*_*i*_ is a set of price volatility, and *i* = 1, 2,⋯, 3765. In addition, *f*(*x*) is a composite function, referring to the four rules designed above. *w*_*j*_ (0 ≤ *w*_*j*_ ≤ 1, and *j* = 1, 2, 3, 4) signifies the weight of above four rules, and *ε*_*t*_ denotes the prediction error.

Forecast mode ②

According to the prediction results of *k*-period price volatility, a following model is established on the basis of traditional time series forecasting methods to predict the future (*t* + *k*)-period price data. The forecasting model is as follows:

$$C_{t+k}{}^{*}=C_{t+k-1}+C_{t+k-1}F_{t+k}{}^{*}$$ (5)

where *c*_*t*_ represents the price data at time *t*, *C*_*t*_ = {*c*_*t*−1_, *c*_*t*−2_,…, *c*_*t*−*l*_} denotes a price sequence, *l* means the number of lagged periods, and *k* is the predicted size.

Here Forecast modes ① and ② are called HOPFM. Let *w*_*j*_ = 1, and *w*_*i*_ = 0 (*i* ≠ *j*), where *i*, *j* = 1,2,3,4, then these forecasting models respectively under the conditions of rules I, rule II, rule III and rule IV can be obtained. At the same time, some prediction models based on combined rules I & II, I & III, I & IV, II & III, II & IV and III & IV are obtained in the conditions that *w*_*j*_ = 0.5, and *w*_*i*_ = 0.5 (*i* ≠ *j*), *i*, *j* = 1, 2, 3, 4. Models in these cases will be analyzed and discussed below. In fact, other combination rules can also be defined according to the topological properties of complex networks, which will not be discussed here because of space reasons.

## Results and analysis

### Occurrence time prediction of new nodes

**Fig 5:**
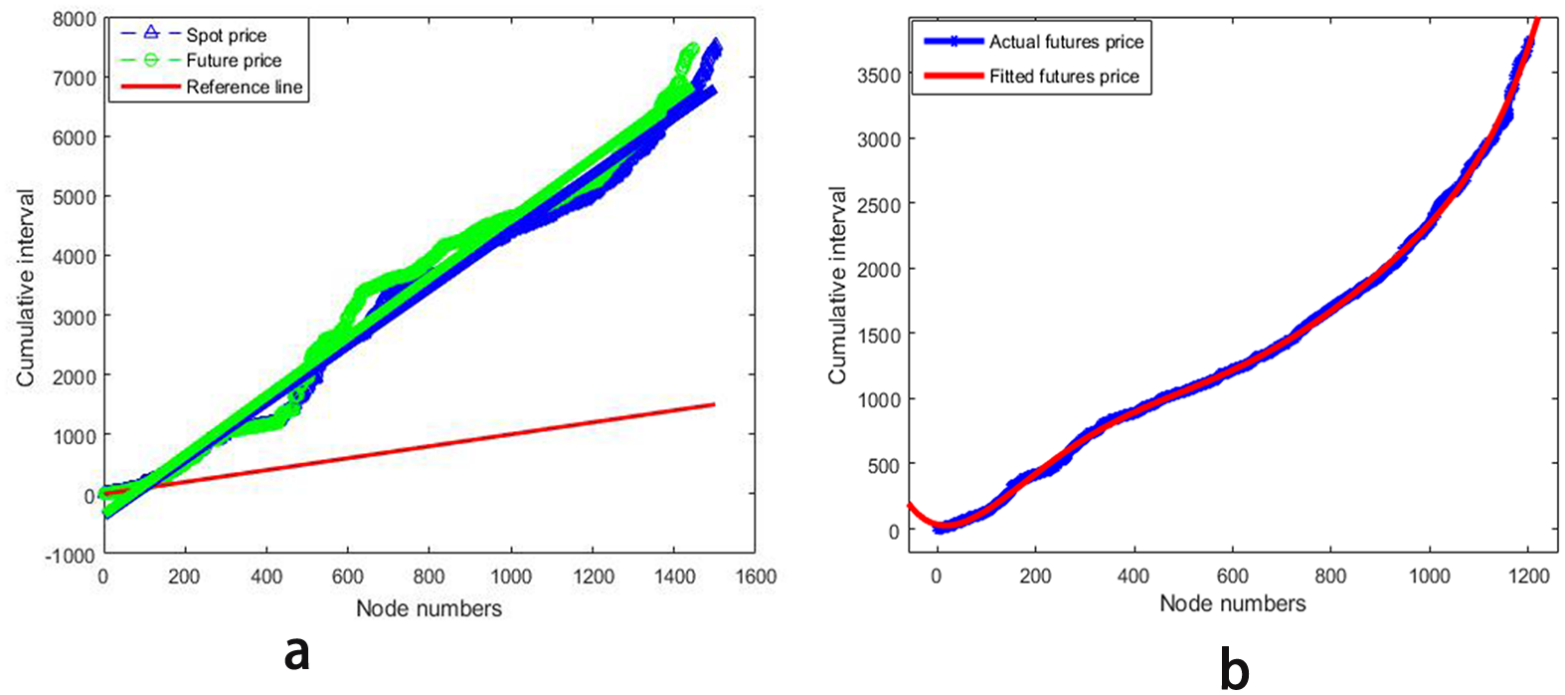
Cumulative time intervals for new nodes. (a) Cumulative time intervals for new nodes in June 2, 1986—July 1, 2016; (b) Cumulative time intervals for new nodes in June 1, 2001—June 10, 2016.

Based on the complex network model established above, we start from the cumulative time interval of new nodes to investigate the emergence law of different nodes. Besides, the results obtained in different time periods are shown in Fig 5a and 5b.

As can be seen from Fig 5a, if the heating oil futures price fluctuation network established is based on 30-year data from June 2, 1986 to July 1, 2016 [41], the cumulative time interval of new nodes as a whole shows a linear growth trend, and corresponding regression equation is *y* = 4.967*x* − 340.11, where 0.981 is the value of its trend line correlation coefficient *R*^2^. However, the cumulative interval of new nodes will not be strictly subject to a linear growth if this time interval is subdivided. Therefore, on the premise of guaranteeing price data are sufficient and results show regularity, 15-year price data from June 1, 2001 to June 10, 2016 are selected as a research object, namely a training set, which is shown in Fig 5b. After trying a variety of fitting methods, it has been found fitting effect of the Fourier function with 3 terms is the best, and its expression is as follows:

$$\begin{array}{l} f(t)=a_{0}+a_{1}\text{cos}(wt)+b_{1}\text{sin}(wt)+a_{2}\text{cos}(2wt)+b_{2}\text{sin}(2wt)+\ldots +a_{n}\text{cos}(nwt)+b_{n}\text{sin}(nwt)+\ldots \\ \;=a_{0}+\sum_{n=1}^{\infty }\left[a_{n}\text{cos}(nwt)+b_{n}\text{sin}(nwt)\right] \end{array}$$ (6)

where *a*_0_ = 4.204*e* + 06, *a*_1_ = −5.774*e* + 06, *b*_1_ = −2.622*e* + 06, *a*_2_ = 1.693*e* + 06, *b*_2_ = 1.951*e* + 06, *b*_3_ = −4.271*e* + 05, *w* = 0.0007076, and with a value of 0.993 for its trend line correlation coefficient. This statistic measures how successful the fit is in explaining the variation of selected data, a value closer to 1 indicates a better fit. In addition, *SSE* = 7.332*e* + 05, which means the sum of squares due to error, measuring the deviation of responses from the fitted values of the responses, and a value closer to 0 indicates a better fit. Experiments show the model established by this prediction method has high reliability and its prediction accuracy is controllable in the specified confidence interval.

Thus, by the Fourier equation obtained by fitting, the cumulative time intervals of new nodes in the next stage can be predicted and then the emergence time of these new nodes is obtained, which is as shown in Table 2.

**Table 2:** Comparison of the occurrence time of new nodes.

No. Type	Actual results		Prediction results of Fourier 3		Prediction results of the NAR neural network	
	Cumulative time	Occurrence time	Cumulative time	Occurrence time	Cumulative time	Occurrence time
1203	3762	2016/6/7	3768	2016/6/13	3681	2016/2/9
1204	3779	2016/6/30	3780	2016/6/29	3682	2016/2/10
1205	3791	2016/7/19	3791	2016/7/15	3728	2016/4/18
1206	3828	2016/9/9	3803	2016/8/2	3741	2016/5/5
1207	3831	2016/9/14	3814	2016/8/17	3839	2016/9/26
1208	3836	2016/9/21	3826	2016/9/2	3854	2016/10/18
1209	3865	2016/11/2	3838	2016/9/21	3857	2016/10/21
1210	3866	2016/11/3	3849	2016/10/6	3858	2016/10/24

**Fig 6:**
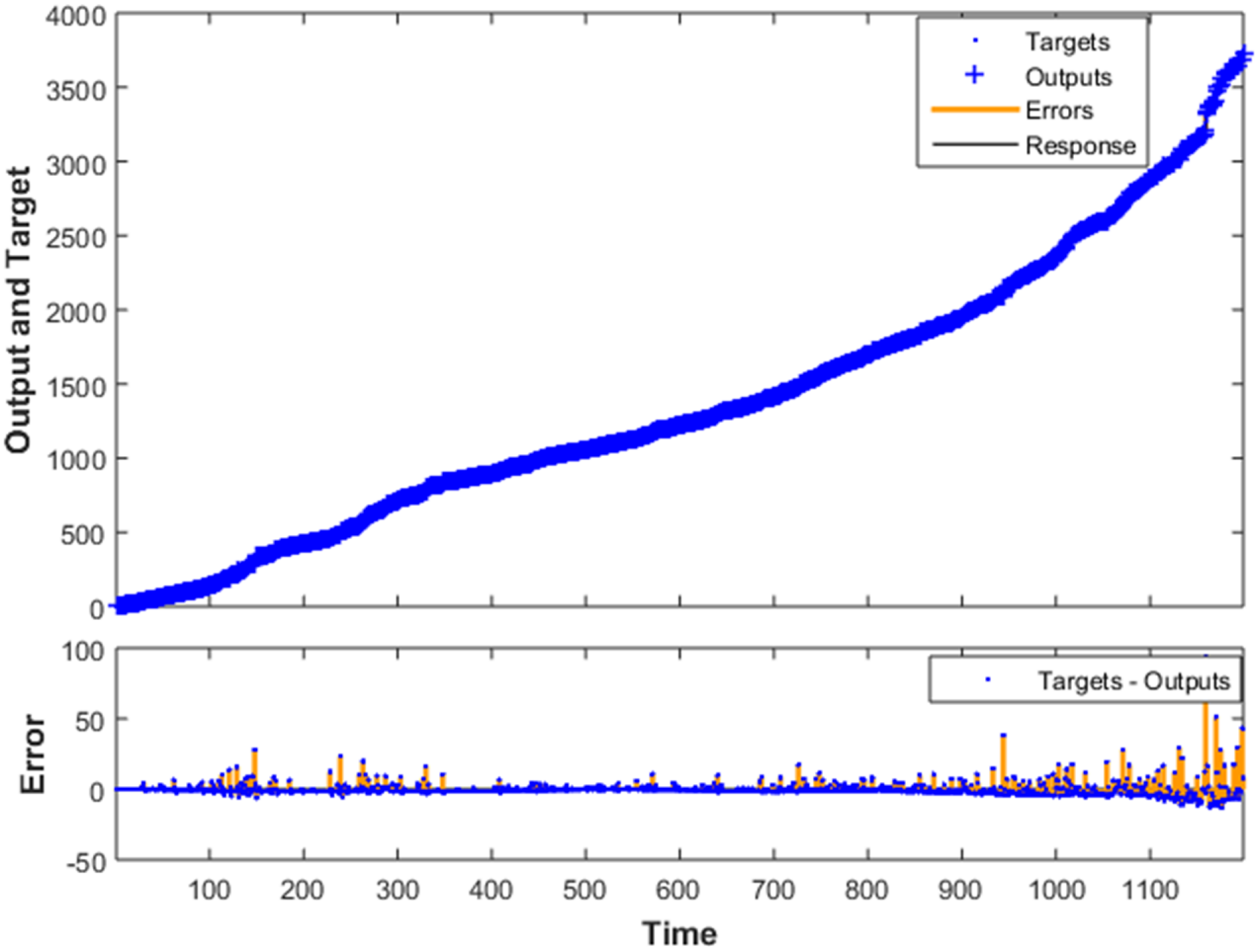
Predictive effects and errors of the NAR neural network.

Table 2 presents the cumulative time interval and corresponding occurrence time of the 1203^rd^-1210^th^ new nodes predicted by the Fourier Model with 3 terms and obtained by actual updated data. In order to facilitate the comparison of its prediction accuracy, Table 2 and Fig 6 also show some prediction results of the NAR neural network, that is, 1226 different nodes are obtained based on the usage of these data from June 1, 2001 to April 28, 2017. Here only the cumulative time interval and emergence time of the 1203^rd^ to 1210^th^ new nodes are given because of a limited space. Furthermore, it can be seen from Fig 6 that the errors as a whole are in [−5,5] interval except for individual points, indicating that the NAR neural network model established and the real relationship between variables are basically the same, which can be utilized to predict this kind of time series. However, Table 2 presents the effect predicted by the Fourier Model with 3 terms is better than that of the NAR neural network method in terms of predicting occurrence time of new nodes, namely closer to the actual date, especially with a high credibility in short terms. Therefore, we will prefer to use this method to predict the cumulative time interval for new nodes in a short period of time.

### Prediction of node names

#### The accuracy evaluation index of HOPFM

The purpose of forecast is to predict the future. Are these predictions accurate? This requires defining relevant criteria for measuring the accuracy degree of prediction. These criteria for measuring accuracy of prediction can account for the overall predictive situation and quality of a forecasting method.

In order to evaluate the performance of HOPFM in futures price forecasting of heating oil, the average absolute percentage error (MAPE) and the root mean square error (RMSE) [41] are adopted in this paper, which are two commonly used indicators of predictive accuracy of evaluation models, giving greater weight to data that produces larger errors. In other words, they are "pessimistic" or "conservative". Their formulas are as follows:

$$MAPE=\frac{1}{N}\sum_{t=1}^{N}\left|\frac{e_{t}}{c_{t}}\right|\times 100\%$$ (7)

$$RMSE=\sqrt{\frac{1}{N}\sum_{t=1}^{N}e_{t}{}^{2}}$$ (8)

where $e_{t}=c_{t}-c_{t}{}^{*}$ is the prediction error, *c*_*t*_ and $c_{t}{}^{*}$ (*t* = 1, 2,…, *N*) denote the true and predicted values at *t*, respectively. Besides, *N* represents the data length of a training set. In fact, the smaller the values of MAPE and RMSE, the better the performance of a prediction model under ideal conditions.

#### The evolution process of prediction accuracy with prediction length

**Fig 7:**
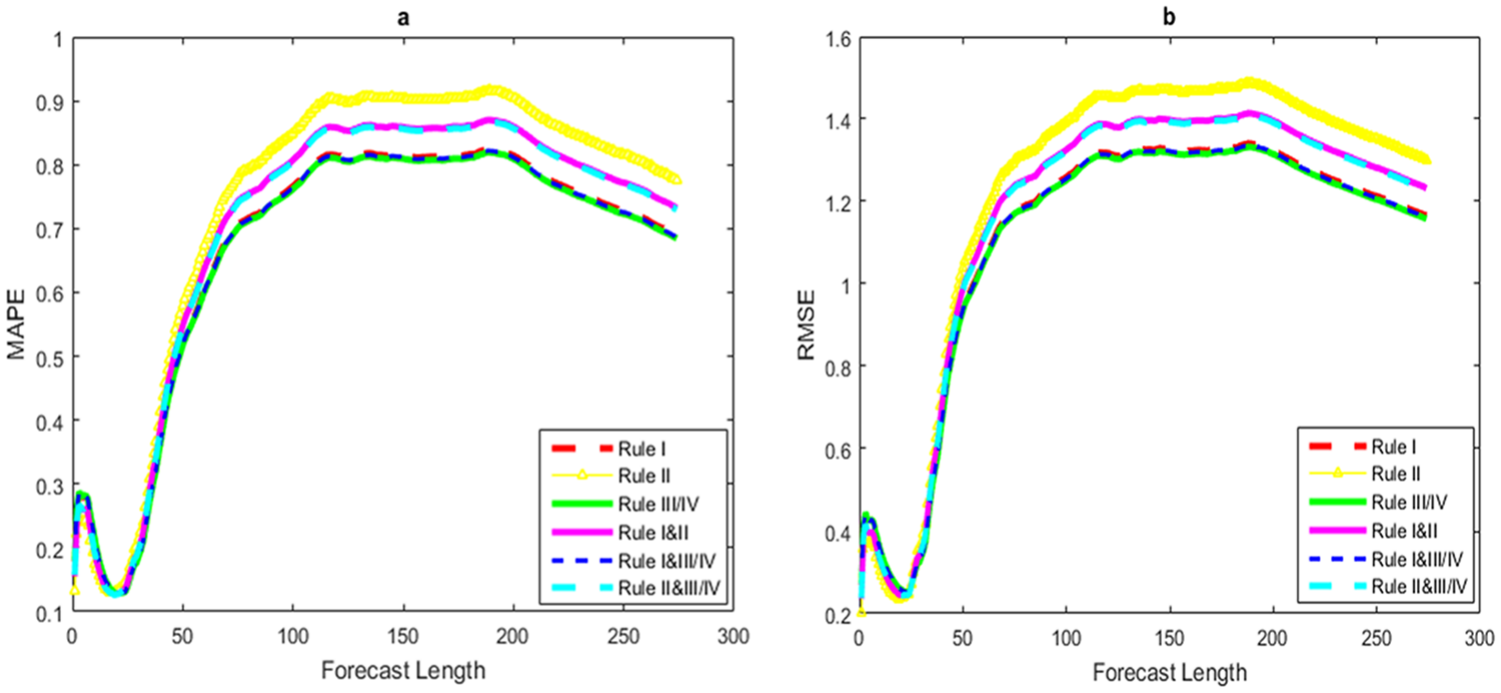
Evolution images of MAPE and RMSE at different predicted lengths. (a) Evolution images of MAPE at different predicted lengths; (b) evolution images of RMSE at different predicted lengths.

As can be seen from above, there is a need for data segmentation before establishing a forecasting model, namely splitting time series data into training sets and validation collections so as to avoid the problem of over-fitting. Afterwards, the price forecasting model is established based on the training set, and then the verification set is selected to measure the prediction accuracy as well as evaluate the predictive performance of this model. Therefore, on the basis of extracting valid information of the past, the prediction accuracy of this model depends on whether the function selection of the effective information synthesis is reasonable or not, it also relies on a selected prediction length, which affects the model we will use for short-term forecast or long-term forecast can achieve higher accuracy of forecast results to some extent. Hence this paper selects daily futures price data of heating oil from June 1, 2001 to June 10, 2016 as a training set, while the data of this period from June 11, 2016 to July 18, 2017 as a validation set, and the evolutionary relationships between the predicted accuracy and predicted length of HOPFM under several design rules are given, as shown in Fig 7.

It can be seen from Fig 7 that both two prediction accuracy indicators MAPE and RMSE of HOPFM under several design rules show a trend of rising first and then decreasing, then rapidly increasing to a certain extent and tending to be stable when dropping to the lowest point, and then facing a slight decline, which shows the prediction accuracy of HOPFM does not improve with the increase of predicted lengths. But instead, there exists obvious superiority in the short-term prediction, especially when the predicted length is 24, namely the forecast date is July 15, 2016, and the values of MAPE and RMSE are respectively close to 0.1 and 0.2 at this time. However, the prediction accuracy of HOPFM under different design rules is different as a whole. Fig 7a shows that when the forecast length is 1–10, average values of MAPE obtained from the prediction model HOPFM under six design rules of I, II, III (III and IV get the same result, so only the rule III is considered below), the combination of I and II, the combination of I and III, as well as II and III of combination are 0.2537, 0.2141, 0.2560, 0.2338, 0.2549 and 0.2350, respectively, indicating that the predictive accuracy of HOPFM under the above-mentioned six rules conditions are ranked from high to low as the II, I & II, II & III, I, I & III and III; average values of MAPE getting from HOPFM of six design rules are respectively 0.1451, 0.1406, 0.142, 0.1396, 0.1457 and 0.1397 when the prediction length is 11–24, which indicates the descending sequence of forecasting accuracy of HOPFM are I & II, II & III, II, I, I & III and III under six design rules conditions; mean values of MAPE obtained by HOPFM under the above six kinds of design rules are separately 0.6086, 0.6762, 0.6051, 0.6414, 0.6068 and 0.6393 when the predicting length is of 25–120, with whose prediction accuracy from high to low ranking rules for III, I & III, I, II & III, I & II, II; mean values of MAPE obtained by HOPFM based on the above six design rules are respectively 0.8152, 0.9054, 0.8104, 0.8600, 0.8128 and 0.8573, with a predicting length of 121–200, it shows that the forecast accuracy of HOPFM under different rules from high to low followed by III, I&III, I, II&III, I&II, II; Finally, when facing the 201–274 forecast length, mean values of MAPE for HOPFM under the above six design rules are 0.7465, 0.8354, 0.7416, 0.7907, 0.7440 and 0.7880, respectively, which shows that under the index MAPE, III, I & III, I, II & III, I & II, II are the order of the prediction accuracy from high to low for HOPFM.

Similarly, when predicting length of 1–10, it can be seen from Fig 7b that the average RMSE of HOPFM under the above six kinds of design rules respectively are 0.3935, 0.386, 0.3967, 0.3660, 0.356 and 0.3675, which turns out that the predictive accuracy of HOPFM under different rules conditions from high to low are II, I & II, II & III, I, I & III and III; when the prediction length is 11–24, average values of RMSE getting from HOPFM based on six design rules are 0.2791, 0.2523, 0.2810, 0.2639, 0.2801 and 0.2646, respectively, which indicates the descending sequence of forecasting accuracy of HOPFM are II, I&II, II&III, I, I&III and III under six rules conditions; when faced with a predicting length of 25–120, mean values of RMSE are separately 1.0288, 1.1364, 1.0230, 1.0823, 1.0258 and 1.0790, indicating that according to the priority order of prediction accuracy of HOPFM, II, I & II, II & III, I, I & III and III are in turn for appreciate rules; when the prediction length is 121–200, average values of RMSE obtained by HOPFM under the circumstance of six design rules are respectively 1.3263, 1.4702, 1.3184, 1.3982, 1.3223 and 1.3939, which shows the forecast accuracy of HOPFM under different rules from high to low followed by III, I & III, I, II & III, I & II, II; average values of RMSE getting from HOPFM under the above-mentioned six rules condition are separately 1.2381, 1.3784, 1.2304, 1.3082, 1.2342 and 1.3040, showing that the prediction accuracy of HOPFM from high to low are III, I&III, I, II&III, I&II and II in turn under different rules.

In summary, we can see that this forecasting model HOPFM based on the price fluctuation network of heating oil exhibits a certain regularity about its prediction accuracy with the forecast length. For one thing, the short-term forecasting effect of HOPFM is much better than that of long-term prediction as a whole. For another, from the different forecast lengths perspective, when the length of forecast is 1–10, the overall prediction effect of HOPFM under designed rules II and I & II is better than that under other rules; when the prediction length is 11–24, the overall forecast effect of HOPFM under that rule I & II condition is the best. While when faced with a predicting length of 25–120, opposite results are obtained under these two indicators MAPE and RMSE, in which case we make a compromise to consider the designed rules I and II&III to be substituted into HOPFM. Besides, the overall predictive effects of HOPFM under rules III and I & III conditions are significantly better than that under other rules when the forecast length is 121–274. Therefore, according to different predicting lengths, choosing appropriate rules into HOPFM will greatly improve the prediction effect of this forecasting model.

#### Predictive analysis of node names under several design rules

There was a large number of literature to predict the price of heating oil previously. In fact, people tend to pay more attention to the trend of data volatility for many prediction problems, that is, a sharp or steady rise, a sharp or steady decline and so on. Since we have utilized five symbols *G*, *g*, *s*, *f* and *F* to represent five states of sharp rise, stable rise, stability, stable decline and severe decline, respectively. Then we predict the names of nodes appearing in such a period of before the 1203^rd^ new node but after the 1202^nd^ new node, based on some prediction results of HOPFM under several design rules and a coarse-grained process of data, and try to compare the influence of several design rules on prediction accuracy of HOPFM, then further analyze the volatility state of heating oil futures prices during this period.

**Fig 8:**
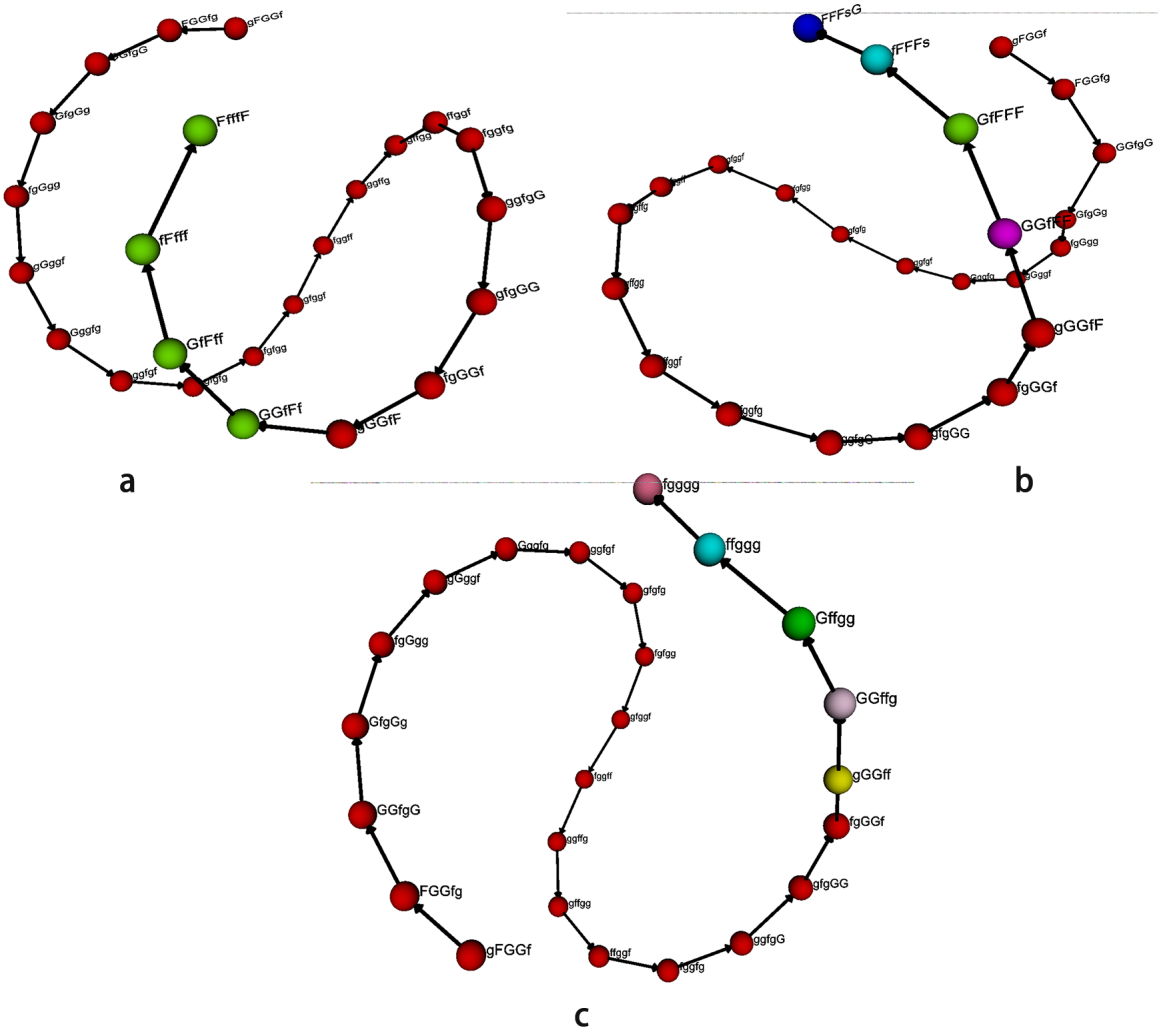
The names of nodes and their transformation relationships in several cases. (a) The names of nodes and their transformation relationships obtained under the actual updated data; (b) the names of nodes and their transformation relationships obtained under the forecast of HOPFM; (c) the names of nodes and their transformation relationships obtained under the forecast of NAR neural network.

Based on these 1202 nodes generated from the price data of heating oil from June 1, 2001 to June 10, 2016, the period of May 6, 2016-June 12, 2016 is selected as a forecast interval of price fluctuation modes according to the forecast results for the appearance time of new nodes. Among them, we only consider the working days during this period, with a total of 24 data that resulting in a total of 24 nodes. Moreover, names of these nodes and their transformation relations in several cases are shown in Fig 8.

For the convenience of comparative analysis, Fig 8a gives the names of nodes and their transformation relationships obtained under the actual updated data. Furthermore, the node names and their transformation relationships predicted by HOPFM and the NAR neural network method are shown in Fig 8b and 8c, respectively. As illustrated in Fig 8b, when the forecast range of fluctuation modes is May 6, 2016-June 12, 2016, the futures prices of heating oil have experienced a total of 24 conversion of volatility states, among which HOPFM successfully predicts the first 20 times, indicating that the accuracy rate of HOPFM for heating oil price fluctuations can reach 83.3%, and the results obtained are consistent under several design rules. In contrast to the results obtained in the part of the evolution process of prediction accuracy with prediction length, it can be seen that in terms of forecasting price data, the above six design rules affect the predictive performance of HOPFM, and their performance is slightly different at different forecast lengths. However, these design rules play the same role in promoting the predictive effect of HOPFM for predicting the trend of price fluctuations. On the other hand, it can be seen from Fig 8c that the NAR network predicts the first 19 times in this prediction interval, which indicates the NAR network has an accuracy rate of 79% for forecasting the price fluctuation trend of heating oil. If based on actual updated data, considering this period from the emergence of the 1202^nd^ new node to the 1203^rd^ new node, namely May 6, 2016-June 6, 2016 as a forecast interval, the prediction accuracy of HOPFM at this time can reach100%, while for the NAR network it is up to 95%. Thus, it can be concluded that although the NAR neural network can predict the test data as a whole, its prediction accuracy is less than HOPFM, which shows a better prediction effect on the price fluctuation trend for HOPFM in short-term forecast.

## Conclusions

In many studies of the past, time series prediction is one of the effective ways and means for price forecasting. On the other hand, complex network, as a popular method for energy research, is an effective tool to study the intrinsic topology dynamics of network. How to effectively combine the complex network and time series models to predict price fluctuation behaviors of heating oil is the main content of this paper. In fact, based on the coarse graining processing of 15-year futures price data of heating oil, this paper proposes a new time series forecasting model of HOPFM combined with complex networks, and discusses a forward-looking behavior of short-term futures price volatility for heating oil. In addition, with the rapid development of network science, the link prediction in networks [42] has been widely used, however, which is only to forecast the connection relationship between nodes under conditions that these nodes are known. The novelty of our method lies not only can the occurrence time of new nodes be predicted, but also their transformation relationship in the forecast interval for a period of time before the next new node appears be obtained by identifying those connection relations of the previous nodes. Furthermore, this method shall be applied to many energy price sequences to describe the evolutionary characteristics of these sequences, and to explain the potential dynamics of time series more simply and accurately.

The main work and prospective results obtained in this paper are as follows:

1. In order to transform the time series into a data network, this paper first takes a coarse graining treatment for the futures prices of heating oil from June 1, 2001 to June 10, 2016, with five characters respectively representing five fluctuation states of prices, thereby transforming the price fluctuation sequence into a corresponding symbol sequence, and then constructs the corresponding directed-weighted network model based on that symbol sequence.
2. This paper introduces the Fourier Model to fit the cumulative time interval curve of new nodes appearing in the network so as to estimate the emergence time of new nodes in the next stage, and comes to an conclusion that the Fourier function with three items has the best fitting effect, whose accuracy degree is better than that of the NAR neural network, especially with a higher degree of credibility in short terms.
3. For the sake of predicting the names of nodes that during the period after the occurrence of a node but before the next new node, four design rules are constructed using valid information such as the topological properties extracted from the network to identify the conversion relations between wave modes of the above-mentioned 15 years. Then based on these four design rules and their combination rules, the prediction model HOPFM is established.
4. In order to clarify how several design rules influence the forecasting effect of HOPFM, the evolutionary images of prediction accuracy with prediction length and predictive analysis of node names under different design rules are investigated respectively. It can be found from the analysis that, on one hand, the prediction accuracy of HOPFM shows some of regularity with the change of prediction length, and different prediction lengths will impact the forecast effect of HOPFM on prices to a certain extent. Therefore, choosing appropriate rules into the forecasting model will greatly improve the prediction effect of this model. On the other hand, with regard to the prospect of node names, same results are achieved when the price fluctuation states are separately predicted under several design rules, which shows there is no difference in the forecast effect of price fluctuation models.
5. A comparison analysis with the results obtained by the NAR neural network is made in order to investigate the advantages of HOPFM based on complex networks in predicting node names. Experimental results show that the prediction accuracy of the NAR neural network is lower than that of HOPFM, indicating HOPFM has a better prediction effect on price fluctuation trend in short-term forecast.

Concerning the future research prospect, in terms of the complex network model established in this paper, we may as well try to remove a small number of nodes and examine whether most of nodes in the network are connected. Therefore, robustness of this network shall be studied in the next phase. In addition, this paper sets four design rules of HOPFM based on several topological properties of complex networks, while whether the rules based on some other topological properties of complex networks and different combination rules have better prediction effect is necessary to be explored.
